# Despondency Among HIV-Positive Older Men and Women in Uganda

**DOI:** 10.1007/s10823-012-9178-x

**Published:** 2012-08-29

**Authors:** Stuart Wright, Flavia Zalwango, Janet Seeley, Joseph Mugisha, Francien Scholten

**Affiliations:** 1 Faculty of Social Sciences, University of Sheffield, Sheffield, UK; 2 School of International Development, University of East Anglia, Norwich, NR4 7TJ UK; 3 MRC/UVRI Uganda Research Unit on AIDS, Entebbe, Uganda; 4 London School of Hygiene and Tropical Medicine, London, UK

**Keywords:** Despondence, Depression, HIV and AIDS, Hope, Social isolation, Uganda

## Abstract

Forty people over 60 years of age took part in longitudinal research over the course of a year on the impact of the HIV epidemic in southern Uganda. In this paper we focus mainly on the data from 26 of the 40 who were HIV-positive. While we observed that feelings of depression were frequently experienced by many of the people in our study, the state of ‘being depressed’ was not constant. Participants regularly expressed economic frustration (because of a lack of money to buy food and other commodities including sugar and soap); medical problems (including those related to HIV) as well as old age, the burden of dependents (including concerns about school fees for grandchildren), feelings of sadness and isolation, and a lack of support from others, as well as stigma, whether real or perceived. However, while worries, sorrow and despondent thoughts were reported in many of the interviews across the study, moods fluctuated moving from happiness and hope, to sadness and despair, from month to month. Concerns regarding the psychological wellbeing amongst older people, including those living with HIV and older carers in Uganda deserve greater attention.

## Introduction

Grace (aged 72 years) was in despair, her eyesight was now so poor she was having problems lighting her small lamp—she asked her 14 year old granddaughter to help her but, Grace said, ‘she scornfully laughed at me for my failure to light the lamp’. Later in the interview Grace brought out a photograph of the girl’s father, her dead son, the picture showed him laughing, enjoying a big meal. Grace looked at the picture for a long time and then said that this son ‘will never get off my mind.’

Grace lives in rural southern Uganda, not far from one of the main towns but her home is very isolated down a long bumpy mud road. She lives with her 90 year old husband who is deaf, blind and incontinent and her orphaned granddaughter. Grace does everything in the home, including cultivating the land and raising animals for their food. They get no material help from anyone. She talks often about making arrangements for the death of herself and her husband. She appreciates the monthly visits of the interviewer for our study to her home, because it gives her someone to talk to and share her worries with.

Research from a range of different countries suggests that symptoms of depression are common in older people (Tannock and Katona 1995; Gutzmann 2000; Zeiss *et al.* 1996; Alpass and Nevilla 2003; Cole and Dendukuri 2003). However, as Zanjani and Rowles (2012) have recently observed, the focus on ageing in rural environments has often been on physical access to care and support, while less attention has been paid to the psychological wellbeing of elders. Indeed they note that the perception that ‘“feeling blue” is a normal part of aging’ (2012: 3) may result in the mental health needs of older people not being addressed. Grace, in our story above, faced many challenges in accessing support and she had many reasons to ‘feel blue’ as she struggled to support her husband and herself, she could not control her granddaughter and she continued to grieve for her son. In addition, she was infected with HIV and worried constantly about her deteriorating health and the challenges of accessing care.

Symptoms of depression have been found to be common among people living with HIV in different settings (Benton 2008; Kalichman *et al.* 2000; Leserman 2008; Myer *et al.* 2008; Olley *et al.* 2004; Simbayi *et al.* 2007). Care-givers of people infected by HIV have also been found to suffer from high levels of depression (Pakenham *et al.* 1995; Bor and du Plessis 1997; Hackl *et al.* 1997; Wight 2000; McCausland and Pakenham 2003; Moore *et al.* 2006). In many parts of Africa, it is older people who shoulder a considerable part of the burden of caring not only for those affected by AIDS-related ill-health but also of children left without their parents as a result of AIDS-related mortality (Wilson and Adamchak 2001; Nyambedha *et al.* 2003; Monasch and Boerma 2004; Foster *et al.* 2005; Ssengonzi 2007; Schatz 2007; Seeley *et al.* 2009, 2010). In addition to being carers, however, increasing numbers of older people in Africa and elsewhere are, like Grace, above, living with HIV themselves (Emlet and Poindexter 2004; Emlet 2006; Ssengonzi 2007; Seeley *et al.* 2010; Scholten *et al.* 2011). We might expect, therefore, both as people living with HIV and those who are care-givers, older people might be particularly susceptible to depression (Kalichman *et al.* 2000).

This paper is based on the data from a longitudinal qualitative study of HIV and older people in Uganda, which was embedded within a cross-sectional study with 510 older people living in southern Uganda. Two fifths of those people were HIV-infected and a further two-fifths had cared for or were caring for an HIV-infected child. One-fifth was an ‘unaffected’ control group. These 510 people were surveyed for a study of the direct and indirect effects of HIV. This quantitative survey, which utilised standard measures for assessing depression, found that 23 % of participants had had an episode of depression in the previous year (MRC/UVRI 2011). The design and results of this larger study are described elsewhere (Scholten *et al.* 2011; MRC/UVRI 2011). However, as we describe in this paper, our qualitative findings suggest a more complex picture of mental health among older people in the cohort than this percentage, based on the cross-sectional survey data, suggests. While we observed that feelings of depression were frequently experienced by many of the people in our study, the state of ‘being depressed’ was not constant. This is why we have chosen to use the word ‘despondency’ in this paper; in the sense of being downhearted (‘feeling blue’ in Zanjani and Rowles terminology) rather than the word ‘depression’ which may imply a clinical diagnosis. However, we do not suggest that despondency and depression are not serious issues among this population, indeed concerns regarding the psychological wellbeing amongst older people, including those living with HIV, and older carers, in Uganda deserve greater attention. Mental health issues affect not only the individuals themselves, but also have serious implications for the wellbeing of dependent children—grandchildren and others—with potential repercussions beyond the lifespan of the older people with whom this study is primarily concerned (Foster 2000).

## Background

Grace, as an older woman with HIV, is not unusual in Uganda. HIV is becoming increasingly common among older people: a 2004–2005 national survey in Uganda reported an HIV prevalence rate of 5.8 % among people 50–59 years, with little difference between men and women (compared to 5.4 % infection among people aged 15–49) (Uganda 2006). Less information is available for people over 60 years, although there is evidence that members of this age group are not only growing older with HIV, but are also acquiring the infection in later life. Very limited data exist that provide an understanding of the impact of infection on the lives and general wellbeing of older people, particularly in resource-constrained settings (Seeley *et al.* 2009; Scholten *et al.* 2011).

Leserman (2008) argues that although HIV treatment often focuses on monitoring critical measurements (for example, the fluctuation of numbers such as CD4 lymphocyte count and viral load), the monitoring of psychosocial risk factors such as mental health and past trauma history are also of clinical importance. Disease, stressful life events, and trauma are understood to account for variation in HIV disease course (Antelman *et al.* 2007; Leserman 2008). Leserman (2008: 544) concludes, “psychosocial factors, such as chronic depression and stressful events, can affect clinical and immunological progression of HIV/AIDS”. There has been a paucity of work on this topic among older people, but Heckman and colleagues (Heckman *et al.* 2002) have observed that HIV-infected older people (50 years and over) in the United States with psychological symptoms also reported high levels of HIV-related stress, less support from friends, and reduced access to health care and other services because of HIV-related stigma compared to others without psychological symptoms. Emlet (2006) talks of the ‘double-jeopardy’ of older people living with HIV in the United States, facing not only HIV-related stigma but also ageism; an additional factor that undermines the mental health of older people (Ramashala 2000; Shortt 2001).

## The Setting

This study was conducted in 2009–2010 in one peri-urban and one rural area in southern Uganda. The rural site was in Kalungu District, where in 1989 the Medical Research Council/Uganda Virus Research Institute (MRC/UVRI) established a general population cohort to study the epidemiology of HIV. Annual demographic and serological surveys have been conducted with this population for the past 22 years, resulting in a wealth of data on approximately 20,000 people in 5000 households. People living in the study area are largely subsistence farmers who produce small amounts of cash crops such as beans, bananas and coffee. The majority (70 %) of the population is ethnically Baganda, and there is a large representation of immigrants from Rwanda (15 %). About 4 % of the population is made up of immigrants from Tanzania; a mixture of other tribes makes up the remainder. The main local language is Luganda, which is spoken and understood by all the tribes. The community is predominantly Roman Catholic (60 %) with 17 % Protestant and 23 % Muslim. Just over 50 % of the population is under 15 years old. Most households have less than five acres of land but relatively few households are landless. The peri-urban site was in Wakiso, the district that includes Entebbe, where the MRC/UVRI headquarters is based. The MRC/UVRI has conducted a number of studies in Entebbe and the surrounding areas over the past 20 years. This study drew on people involved in some of the earlier research: for example, a study on strategies to reduce the burden of opportunistic infections. Some participants came from The AIDS Support Organisation (TASO) in Entebbe. The ‘peri-urban’ label refers to the site of service provision, not place of residence. Many participants lived in rural areas around Entebbe and were not, therefore, residents of the town. The population in Wakiso district is more mixed than at the rural research site in Kalungu, because people from many different tribes have settled near Entebbe. While there is a strong Roman Catholic presence in the district many other Christian denominations as well as Muslim groups are also represented. Many people in rural areas near the town still practice cultivation as their main form of livelihood, but there are also people engaged in fishing and various forms of trade, as well as formal employment in teaching, health care, and cleaning services.

The sample for the quantitative survey consisted of people aged 50 years and over. Five groups were selected, each with 100 participants of whom half were living in a rural area (Kalungu district) and half in a peri-urban area (Wakiso). The groups were older people who: have an adult child who died of AIDS; have an adult child who is living with HIV and on antiretroviral therapy (ART); have no child with HIV/AIDS and are not infected with HIV (comparison group); is HIV infected and on ART for at least 1 year; and is HIV infected and not on ART or on ART less than 3 months. In total 510 people were interviewed in the quantitative component: 256 in the rural and 254 in the peri-urban area, 61 % women and 39 % men. The mean age overall was 65 years, but for the HIV-positive groups the mean age was 59. The findings of this study are reported elsewhere (Scholten *et al.* 2011; MRC/UVRI 2011).

For the qualitative study 40 participants (20 from each site) were selected from the quantitative study sample. These participants were aged 60 years and over, in order to focus on people who may be more likely to face the challenges of failing health. Twenty six of these people were HIV-positive and the data from these people forms the main focus of this paper. The 20 rural participants were chosen to faithfully mirror the sample from the quantitative survey sample comprising of the five different groups. However, while eight HIV-positive people should have been included in this sample, the number was only 6 (3 of whom were on ART) because an attempt to get a representative cross-section by age and gender, as well as replacements, slightly skewed the final sample. These 6 HIV-positive people received free care from the MRC/UVRI clinic. There was a larger number of people aged over 60 who were HIV-positive in the peri-urban setting, so the decision was taken to focus on this group in that sampling. Therefore, all 20 peri-urban participants (9 men, 11 women) were HIV positive and receiving free care from The AIDS Support Organisation (TASO) in Entebbe. Fourteen had been on antiretroviral therapy (ART) for at least 1 year, while the remaining 6 were not on ART but were taking cotrimoxazole. A number of these participants had also taken care of children or other relatives who had been sick with AIDS-related illnesses. The 14 HIV-negative participants from the rural area provide for some comparison with the total of 26 HIV-positive participants, 20 of whom were on ART.

Many of the participants in both locations had lost supportive children and grandchildren or were worried about losing their children as a result of the HIV epidemic. A few were reliant on care and support provided by grandchildren for whom they were responsible, resulting in different levels of mutual responsibility, support and care. Some of the older people received support from their own children, now adults, and some were reliant upon neighbours to help them get food and water, although this kind of support was not guaranteed. Invariably, the participants had to do as best as they could to support themselves and, for those with a heavy care-giving burden, their grandchildren.

Of the 40 participants, 17 were aged 60–69 years, 19 were aged 70–79 years; 4 of them were aged above 80 years. Eleven were married, of whom only 1 was a woman; 21 were widowed. Ten participants (6 men and 4 women) lived alone while others lived with their grandchildren or other relatives. Twenty-five of the participants were ethnic Baganda while 10 were of Rwandese or Burundian origin. The remainder were from other tribes.

Seven experienced interviewers (2 men and 5 women, all but 1 aged above 60 years) made monthly visits to the 40 participants over a period of 1 year. The interviewers collected a monthly oral diary of each person’s life focussing specifically on the week prior to each interview. This 7-day recall period provided detail for a period that was still relatively fresh in each participant’s memory. An additional interview was conducted specifically to focus on documenting each respondent’s life history.

The method of interviewing, a semi-structured extended conversation, was designed to provide qualitative insights into changing family situations and relations, older people’s tasks and responsibilities, visitors and visits, diet and food availability, and their feelings about their health and wellbeing and growing older. The interviews also addressed participants’ hopes, concerns, perceptions, expectations and experiences. The resulting oral diaries provided data on the impact of early life experiences, gender differences and perceptions of support systems; as well as a valuable understanding of the relationship between perceived support and participants’ physical and mental health. The diaries were written up from notes by interviewers (tape recorders were not used due to concerns from some participants about recordings) immediately after each interview. The interviews were conducted in Luganda or, in one case Swahili, known to both participant and interviewer and translated and transcribed into English.

In our analysis we are able to cross-reference the qualitative data with data gathered in the quantitative survey because the qualitative sample was drawn from the quantitative sample.

The Science and Ethics Committee of the Uganda Virus Research Institute provided ethical approval for this research, and the Uganda National Council for Science and Technology provided overall clearance. Participants found sick at the time of interviews were referred to MRC/UVRI study clinic.

## Results

### Evidence of depression—quantitative survey findings

While this paper focuses on the findings from the qualitative research, a starting point for discussion on depression and wellbeing is provided by the quantitative data collected from the participants. The quantitative study identified cases of depression according to two methods: a single question, and a series of questions. Firstly, the participants were asked whether or not they had ever been diagnosed with depression. Twenty-two people (4.3 %) in the larger study reported that they had indeed been previously diagnosed with depression (MRC/UVRI 2011). However, this figure excludes any participants who may have experienced depression but were never formally diagnosed. There is a possibility that participants may have chosen not to disclose depression-related information, perhaps due to mental health-related stigma.

Secondly, participants were asked a series of questions about how they felt during the 12 months prior to the survey, to assess the prevalence of depression that had not been formally diagnosed. Questions included: “Have you had a period when you felt sad, empty or depressed?” and “Have you felt that your energy levels have decreased?” The subjective answers to these questions allowed for screening for depression. It was ascertained that a total of 23 % of the 510 participants (comprising all five groups) had experienced an episode of depression during the previous year—a significantly higher number than the 4.3 % who reported that they had previously been diagnosed with depression.

Of the 23 % of participants who had experienced an episode of depression during the previous year, the quantitative study identified a large gender difference, with depression being far more common among women (28 %) than men (16 %) (MRC/UVRI 2011). The study found that there were low levels of depression amongst HIV-positive older people, and that for neither of the HIV-positive groups (those on ART and those not on ART) was HIV significantly associated with depression.

As stated above, the quantitative study assessed the prevalence of depression that had not been formally diagnosed by asking a series of direct questions about how the participants had felt during the 12 months prior to the survey (a single 12-month recall period). Meanwhile, the qualitative research team took a different approach and were concerned with general circumstances and experiences throughout the year of the qualitative study.

While the quantitative study revealed a significant difference between formal diagnosis and actual subjective experiences of depression (4.3 % and 23 % respectively), recounted through a reflection on the previous year, it found no cases of depression amongst the 17 HIV-positive Wakiso participants included in the qualitative study and for whom quantitative data on depression were available. In the rural area, among the 20 qualitative study participants, 9 cases of depression were identified (45 %). These included three men (33 %) and six women (67 %). All these cases were HIV negative and 6 (67 %) were aged above 70 years.

Since the research was primarily interested in the direct and indirect effects of HIV/AIDS and anti-retroviral treatment (ART) on the health and wellbeing of older people, and this paper is, more specifically, concerned with the question of despondency and psychological wellbeing, we now examine the possibility of a link between physical health problems and despondency.

### Evidence of despondency—qualitative findings

The examples used in this section are of HIV-positive participants from both the rural and peri-urban sites. They show despondency and feelings of unhappiness attributed to physical health problems (related to ageing as well as HIV) as well as other factors such as financial problems, isolation, loneliness and lack of support. Significantly, experiences and perceptions of all these factors influenced participants’ subjective explanations of the causes of unhappiness, frustration, despondency and even depression. Self-reported health is one example.

The quantitative study (MRC/UVRI 2011: 19–20) reported that 53 % of participants perceived their own health as “moderately good”, 17 % as “good”, 14 % as “very good”, 15 % as “bad” and 1 % as “very bad”. HIV-positive participants on ART were the least likely to perceive their health as “bad” or “very bad”. Among participants aged over 70, 29 % reported their health as “bad” or “very bad”, compared with 9 % aged 50–59. In the poorest households, 22 % reported that their health was “bad” or “very bad” compared with 13 % in the wealthiest households. In contrast to this cross-sectional view, the longitudinal qualitative study found significant inconsistencies and temporal fluctuations in the self-reported health of individuals over the 12-month research period.

We found that self-reporting of health conditions, as well as financial and livelihood hardships, were filtered through specific age- and culturally-related dispositions, with individuals often making pragmatic comments that seem incongruous when considering health complaints expressed in the same interview. As such, it is important to consider the distinction between actual or diagnosed health problems, and self-reported health. There were often significant differences between how a single participant might experience and perceive their health, report their current health condition, and then ‘rate’ their health in a way comparable to the types of responses captured by the quantitative study. For example, 63-year-old Patricia, who had been treated for tuberculosis years earlier, was receiving cancer treatment as well as ART, and also had eye problems. She walked with crutches and had experienced persistent thigh pain since falling down from the steps at her home in January 2008 but had not reported this to the hospital due to fear that treatment would be expensive and that her property would be stolen by her co-wife’s son in her absence (he frequently stole to pay for alcohol). Despite all of these health problems, when asked how she is feeling within the 7-day period before 5 of the monthly interviews, Patricia said that she rated her health quite highly: commenting that she had not experienced any “serious illnesses” or had no “serious sickness”. She said that ART and cancer treatment had caused a great change in her life and she no longer falls sick frequently, and that she was not affected by her age.

Shifting perceptions and ratings of health were influenced by the general mood (not simply the physical health) of the respondent, which had in turn been influenced by a wide variety of factors: food availability and problems or successes with agricultural work; feeling particularly abandoned by a relative; instances of increased support from others; the number of recent funerals attended or the death of a close relative. An example of the shifting perceptions of health is given by the case of Linda, a 70-year-old HIV-positive participant who lived alone and did not have wider family responsibilities. During her first monthly interview she complained of general weakness, fever, poor appetite and diarrhoea. Then, during the subsequent 4 months she reported a pain in her right leg, “painful muscles without fever”, a cough and flu, and yet she said there was “nothing disturbing” her, that she was doing well with her treatments and had “no serious health problems”. She said that ART had greatly improved her health to the extent that she did not feel that health issues affected her wellbeing.

Other cases contrasted sharply with hers, and illustrate greater levels of despondency and unhappiness attributed to physical health problems by other participants. A 60-year-old woman in Wakiso, named Joyce, who was not yet on ART because her CD4 count was still above the threshold for treatment, had been prescribed cotrimoxazole. This participant reported feeling weak and experiencing physical pains “constantly attacking” her, preventing enjoyment of life and causing insomnia. She thought a great deal about the “deadly disease, which is never going to cure” and she was worried that she may die at any time.

Such feelings did not only occur among women, David, another Wakiso respondent, aged between 60 and 70 years reported that his health deteriorated significantly over the period of the monthly interviews, despite being on ART. During the first interview, he said he was active and had no health problems, but later he reported that bad physical health affected his wellbeing, made him feel unhappy, prevented a “sound mind” and made him “lose peace”.

For many participants, deteriorating health was not necessarily complained about as intrinsically problematic, but as instrumentally problematic, affecting many participants’ ability to do agricultural work, provide enough food for themselves and their households, which in turn emphasised to them their isolation when external support was not available. For example, Joyce, the 60-year-old woman described above, said that weakness and physical pain made her conscious that she was approaching a time when she would be unable to do anything for herself. Although ART might sustain Joyce’s health, if in future she received this treatment, she described her most troubling problem as the daily struggle to “upgrade” her life, and her biggest hope was for more money.

In contrast with the case of Linda (described above), Joyce was the head of a household which usually included up to six people including her daughter (aged 22), grandchildren, nephews and nieces (aged between 3 and 18 years), and Joyce had many associated responsibilities. When a nephew grew old enough to support himself, she was brought another 3 year-old grandchild to look after. Sometimes Joyce received no visitors and no assistance. Although she did report receiving occasional support (food items, for example) from her son and daughter. She said she could not demand anything more than what they gave voluntarily. The support this grandmother received was invariably offset by obligations and the demands of the household, even at the expense of her own wellbeing. One month she reported being unable to spare money for her own health treatment because she had to pay school fees for a nephew. Financial worries and an unsatisfactory diet led Joyce to think a lot about how she would “get rich”.

Joyce worked as a school cook to make an income but had difficulty getting paid; she was also busy with private agricultural activities, but she said that she forced herself to work despite either constant or intermittent sickness or physical pains. A major worry was a lack of a well-established income-generating activity, and these problems, as well as an unsatisfactory diet, were intensifying and created what she described as “mental stress”. While physical health problems affected her ability to maintain agricultural activities, physical health was one of a range of factors that affected her psychological wellbeing.

Significantly, Joyce spent a month in prison in 2008, apparently because she severely punished one of the children she was caring for when the child would not do as she was told. This incident might be an indication of the pressure she felt to maintain the household despite the lack of sufficient financial, practical and emotional support, and her inability to cope with her responsibilities. She said, “I suffered in prison”; the interviewer noted that the incident had “left a scar on her heart”.

David, the 60-year-old Wakiso participant on ART (introduced above), whose physical health affected his wellbeing and made him feel unhappy, explained that he had to force himself to do agricultural work each day despite bad health in order to earn money and to eat. Sometimes he only had strength to work for an hour, or not at all. Financial problems and loneliness, specifically the need to be financially better off in order to find a wife to look after him, were recurring topics of conversation and a cause of insomnia. David was frustrated because people took advantage of him and did not pay him enough, which, the interviewer noted, is “bringing sorrow to his heart”. This participant consistently fretted about how he lacked money to “get a woman who would make him happy”.

David lived alone with no family and said that living alone reduced his happiness, and loneliness affected his health, especially when his landlady was away. The landlady, who lived in an adjacent dwelling and neighbours provided some assistance when David was sick, but not when he was well. He said, “There is no one who I can refer to for help”. He said he was less lonely when people visited and chatted with him for a little while, but many villagers greeted him and continued without stopping for a conversation. Describing his own circumstances by using the metaphor of being eaten by *namuginga*—a pest that eats a sugar cane from the inside while the cane continues to appear intact outside—David seemed very uncomfortable with the awareness that both his poverty and HIV status were obvious to others. This awareness affected his confidence and self-esteem.

A number of participants hoped that ART would transform their lives for the better. For example, the case of Vincent, a 65-year-old fishmonger who was also busy with private agricultural activities. In his first monthly interview, Vincent reported that he experienced backache despite medicine from TASO (cotrimoxazole, not ART). However, backache was not mentioned again in any subsequent interview. In the second month he reported that his condition was okay, he had gained weight and his leg pain had reduced. In the third month Vincent reported swollen legs, pain from his knees down to his feet, and general weakness preventing him from doing as much work as he would like. He said this was affecting his wellbeing. Other reported health problems included small, itching and painful spots on the skin, high blood pressure and “terrible flu”. Vincent relied on a walking stick and felt bad when friends were moving at high speed but he could not; he said he had “taken all kinds of drugs” for leg pain, but to no avail, and wished the doctors could put him on ART because “maybe my legs would improve”.

Vincent moved between two homes and two wives who looked after him (cooking, washing and ironing); some children lived in both homes. Compared with other male participants who lived alone and regularly said that they wanted to find a wife to look after them and to reduce their loneliness, Vincent was not lonely and isolated. However, deteriorating health made his responsibility for two families a considerable burden. Vincent’s wellbeing was affected by physical health problems indirectly, in that health problems were an obstacle to work, which in turn compromised his income and created frustration. However, Vincent did seem to be faring better economically than many other HIV-positive participants. He made two seemingly contradictory statements in one interview: on the one hand, he said that there were “no marked problems” and he “hardly gets any hardships” because he had money and a good life. On the other hand, he said he was depressed because of business problems and because workers had stolen money. Vincent’s “depression” seems to have been a symptom of frustration about his financial situation. He has made many preparations in the hope that his family will survive economically in the event of his death, but believed that his money “will never be enough”. He said he was afraid of dying and leaving his two wives, children and relatives. Vincent said that all his complaints were “pointing to poverty” and that HIV was “becoming a stumbling block”.

Some participants did not seem to suffer from unhappiness and despondency as a direct result of their physical health problems. Rather than attributing diet and financial problems to poor health, they attributed poor health to economic and livelihood circumstances. For example, throughout the interview period, a 73-year-old participant named Moses experienced “more or less continuous backache” as well as acute pain or numbness all over his body, including his knee and elbow joints, feet, headaches, and “a sharp pain on the left hand near the armpit”. The only exceptions were in month 2, when he said he was generally feeling well because of cotrimoxazole, and in month 7, when he described his physical health as good despite reporting heaviness in his back and the problems of living with HIV. Moses considered food to be a primary medicine, as hunger may have been causing him pain. Lack of food reduced his strength and the effectiveness of medicines, he said:

“[T]he most important thing that pains me and disturbs my mind most is the diet, because if I had a diet which was good I would feel healthier than I do now. […] This continues from day to day with no hope of having somebody to ensure that I feed better”.

Moses was owed money, an on-going problem with no resolution in sight, and which caused a great deal of worry and frustration, and contributed to insomnia. He said that he hoped he would feel very happy when he was paid his money. He tried to support himself through his rental property and vegetable cultivation but said his life pattern and lack of money were not at all conducive to good health and wellbeing. He did not have regular meals due to a scarcity of money and because he sold *waragi* (alcohol), so people were always coming and going, preventing any kind of routine. He shared his home with a friend who stole his *waragi* and became abusive to him and also the neighbours.

Although Moses did not drink heavily and had tried to avoid drinking, he said he drank in moderation because it allowed him to socialise with others, prevented loneliness, and helped him sleep. Sometimes he experienced insomnia; at other times, bad dreams troubled his mind. The dreams included money-related scenarios. It would appear that financial circumstances were a greater source of anxiety and uncertainty than his physical health problems.

For a 73-year-old HIV-positive participant named Agatha, isolation and anxiety over a lack of practical and emotional support were the main causes of unhappiness and despondency. In terms of health, Agatha experienced headaches as well as leg and feet pains, a cough, palpitations, ulcers, epistaxis, toothache, severe neck pain, and an unspecified blood pressure issue. Throughout the research period, she fluctuated between feeling fine for a few days and feeling sick for a few days (expressed on a separate occasion as: “one week she is fine and the other she is sick”). However, Agatha seemed to belittle her health problems, rather than feeling unhappy. She said she does not get sick often, that her life was fairly good, her condition is good, and she felt strong enough to care for herself.

Although Agatha sometimes complained of difficulties with household activities due to old age, leg and foot pains, at other times she was able to walk as far as 6 km and was able to work in her garden. She seemed to lament her changing health and increased difficulty with household activities with a degree of pragmatism (“I have to face the present situation and adjust my life”) and described herself as a very strong woman who stands whatever comes her way.

In this way, the daily difficulties of living with health problems did not seem to be the cause of any despondency, especially compared to the insights provided by discussions on other topics. Agatha did experience psychological problems—apparently she is “always worried and is in thought” regarding her last days and years, although this anxiety was about loneliness, isolation and lack of support, rather than being directly associated with her health.

Agatha seemed quite isolated and did not receive visitors. Passers-by always “branch off to greet her” but did not stay long. She described herself as a very social person, and indicated good relations with the community, but she felt out of place at social gatherings because she thought of her condition “every now and then”. She said she did not associate much with village members because they gossiped a lot; however, she described herself as a ‘consultant’ for all those who want to know about HIV/AIDS in the village.

In some cases, the depiction of the causes of despondency and unhappiness was more mixed, even contradictory. Judith, a 67-year-old HIV-positive participant in Kalungu, had been married twice but had no children and lived alone with nobody to assist her. At the start of her participation in this study, Judith received visitors that came to buy alcohol and cigarettes most days; however, after a while she stopped selling alcohol and cigarettes because customers failed to pay her. Also, Judith experienced violence: for example, a drunk male customer assaulted her, leaving her to crawl to her room while others laughed at her; the assault resulted in a back injury and on-going back pain which caused her to lose weight and bend while walking. The interviewer noted that Judith looked “as if she wanted to cry”.

When diagnosed as HIV-positive, Judith believed that it was her “time to die”, despite being prescribed ART, because there was no actual cure. She said she had no trusted friend to share the test results with, and that she feared people would “back bite” her when they come to know her status. Similar to David (described above), who seemed very uncomfortable with the awareness that both his poverty and HIV status were conspicuous, Judith said that most of the time her clothes were clean, despite difficulties, but that she was “fearing a rumour to ‘snick’ about her [if] found with dirty clothes”. However, on another occasion, she said that she did not fear even when the community knows that she is HIV-positive as long as she receives treatment.

Despite her poverty, Judith had a pragmatic faith and seemed to draw much strength and hope from both religion and her health carers. She said that when she was diagnosed as HIV-positive, she “promised to stick to God”; she expressed happiness that she was treated after diagnosis, and that ART had improved her life. She said she was ready for death, which is unavoidable for all human beings whether rich or poor, old or young—“even the Pope in Rome”. She added that she felt no fear of becoming old and prayed to stay for 40 more years. However, there was another side to Judith’s moods and sense of wellbeing. Once, on the verge of tears, she said:

“It was very bad for me not to have a child; otherwise the child could assist me and take care of me. There came a certain time when I wanted drinking water and nobody was around; it took time for me to get out of bed, I crawled, which showed me that I was almost at a point of death and needed help!”

Judith used a walking stick to move even from her room to the kitchen to prepare meals. She said that her life had “become weak” and that she had “stopped agriculture activities long ago”. On one occasion, Judith reported having spent a whole day crying because she had no food or even tea. Yet, showing how circumstances can change, in month 12, she said she faced no food-related hardship because friends supported her; only the ‘sauce’ (vegetable or meat to go with a starch) was her problem, as she had no money to buy fish or meat. The interviewer noted: “She almost cried while saying that”.

Worries over food affected everyone at some point during the study. Most of the older people depended on one meal a day; others survived on one meal for two or more days. Sometimes when participants said they eat one meal a day, they also ate leftovers for supper or breakfast but the quantities were small and they did not consider this to be a meal. There were also seasonal variations: poorer people experienced exacerbated food shortages for 1 or 2 months each year during the dry season. However, during the study period, the dry spell lasted 3-4 months. Fatuma, a 73-year-old Kalungu participant who lived alone and was HIV positive, said that food was a constant problem. She was entirely dependent on the little that was available from her garden, and only managed to have one meal each day (supper) plus maize porridge for breakfast and lunch. She faced particular hardships in getting food during the dry season, and she said that digging cassava from the dry soil pained her very much. Eighty-year-old Isaac who worked as a cobbler at a trading centre, said he used his meagre earnings to buy meals from a local hotel and, therefore, ate only one meal each day. He liked to go to the trading centre to find people to talk to, and sometimes people around the trading centre fed him when he had no money, which he appreciated.

Although some participants regularly complained about health matters, caused by old age as well as HIV, the main factors that negatively affected wellbeing and caused despondency were interrelated problems and worries relating to finances, work, diet, other resource constraints, lack of assistance and support, loneliness, isolation and separation from important relatives. These same factors affected participants who were not HIV-positive. Madina, for example, an 80 year old participant from Kalungu, lived alone in a poorly thatched mud house, and struggled to get enough food to eat. Her daughter had died of AIDS-related illnesses, leaving her to the mercies of her grandson, who, she complained, failed to help her and was waiting for her to die.

Of the 40 participants, 26 of them said that their health problems had become an obstacle to their livelihoods, food availability and in turn their wellbeing in general. The transcripts of interviews with 11 participants indicate that their health did not have a significant impact on livelihoods and food availability. The differences between the 26 and 11 participants did not appear to be due to bad or good health, gender, or HIV-status, but were often determined by regular family support, whether or not the participants lived in large households or alone, whether or not they were solely responsible for an extended family of children. It was clear that without family support, many of those 11 participants would find that their health severely hindered their ability to grow food or earn even a small cash income.

## Discussion and Conclusion

The quantitative study identified factors significantly associated with depression, including socio-demographic factors (gender, wealth, education levels), medical illnesses (chronic back pain, diabetes and visual problems), biological markers (grip strength), and psychosocial factors (social network index and WHO Disability Assessment Schedule). However, the qualitative study found that the psychological wellbeing of many participants, who apparently were not depressed according to the quantitative study, seemed to be adversely affected by some of these socio-demographic, medical, and psychosocial factors. Many of the participants regularly expressed economic frustration (because of a lack of money to buy food and other commodities including sugar and soap); medical problems (including those due to HIV) as well as old age, such as severe and ongoing backache, and decreased energy levels resulting in an inability to produce enough food; the burden of dependents including concerns about school fees for grandchildren, feelings of sadness and isolation, and a lack of support from others, as well as stigma, whether real or perceived. However, while worries, sorrow and despondent thoughts were reported in many of the interviews across the year of the study, moods fluctuated with happiness and unhappiness intermingled in many narratives often when older people reflected on the challenges they faced and then, later, recounted the visitors or help that they had been given.

Social isolation has been identified as a risk factor for depression in older people in other studies (Gutzmann 2000; Findlay 2003; Alpass and Neville 2003). This isolation is caused not only by reduced mobility but also by ‘the inevitable loss of family and friends’ (Alpass and Neville 2003: 212). In our study the loss of their peers was compounded for some by the loss of their children to AIDS-related illnesses. The acute pain of the loss of a child has been documented in a number of different studies (Cobb 1956; Rubin 1993; Videka–Sherman 1982) and attention has also been paid to the particular experience of older parents, like Grace in the story at the beginning of this paper, grieving the loss of an adult child (Moss *et al.* 1986; Rando 1991; Cacace and Williamson 1996). Children are an investment in the future and in a very real sense in countries such as Uganda with no social security system, a safety net for the parent. It is not surprising that the death of a child should lead to feelings of despondency and despair because that death has eroded ‘hope’.

Hope is an important concept in our study. Dufault and Martocchio (1985) define hope as “a *multidimensional* dynamic life force characterised by a *confident* yet *uncertain* expectation of achieving a *future* good which, to the hoping person, is *realistically* possible and *personally significant*” (p. 380, italics theirs). The participants seemed to look to the future, to be able to cling on to hope, even when their circumstances seemed dire. For example, the loss of Grace’s only son and the bad behaviour of her granddaughter drove her to despair but at another time Grace confided that she hoped her granddaughter would change and be more helpful when she grew up. She also hoped, ironically, that her elderly husband would be found to be HIV-positive (he had not been tested) because this would give her access to free medical care for him.

For many participants who were on ART and went to clinics for regular check-ups and had contact with a counsellor, their feelings of social isolation seemed to be moderated by this social contact. ART has been shown to have a dramatic effect on the health and well-being of people living with HIV, literally restoring their hope of life (Russell *et al.* 2007) but for the older people in our study ART also gave access to people to talk to and share with, and invaluable sources of support. It would appear that access to care and support made the HIV-positive older people less susceptible to depression than their HIV-negative peers, a finding corroborated by other recent research (Nyirenda *et al.* 2012).

Not surprisingly, the interviewers came to play a part in the participants lives, serving for some as a much needed visitor who was prepared to give them time to talk and reminisce; perhaps helping to alleviate feelings of despondency and isolation. The required social support that lifted the mood of older people in this study population was very often ‘someone to stop and chat’, getting enough to eat and someone to sympathetically treat their many ailments.

Attention to the mental health of older people needs to take account of what Schatz and Gilbert (2012) have recently highlighted as the social reality of these people’s lives. They have called for a greater ‘emphasis on a context specific and nuanced understanding’ of ageing (p.23). The in-depth analysis presented in this paper has demonstrated the importance of context for understanding despondency among older people in general and, related to our findings, among those who are living with HIV. The psychological wellbeing of many older people in Uganda is intimately bound up in the struggle with poverty that they face on a daily basis in their lives.
